# Global transcriptome analysis reveals resistance genes in the early response of common bean (*Phaseolus vulgaris* L.) to *Colletotrichum lindemuthianum*

**DOI:** 10.1186/s12864-024-10497-7

**Published:** 2024-06-10

**Authors:** Yujie Chang, Yonghui Liu, Lanfen Wang, Shumin Wang, Jing Wu

**Affiliations:** Key Laboratory of Grain Crop Genetic Resources Evaluation and Utilization, Institute of Crop Sciences, Chinese Academy of Agricultural Sciences, Beijing, 100081 China

**Keywords:** Common bean, Anthracnose, Resistance gene, RNA-Seq

## Abstract

**Background:**

Disease can drastically impair common bean (*Phaseolus vulgaris* L.) production. Anthracnose, caused by the fungal pathogen *Colletotrichum lindemuthianum* (Sacc. and Magnus) Briosi and Cavara, is one of the diseases that are widespread and cause serious economic loss in common bean.

**Results:**

Transcriptome analysis of the early response of common bean to anthracnose was performed using two resistant genotypes, Hongyundou and Honghuayundou, and one susceptible genotype, Jingdou. A total of 9,825 differentially expressed genes (DEGs) responding to pathogen infection and anthracnose resistance were identified by differential expression analysis. By using weighted gene coexpression network analysis (WGCNA), 2,051 DEGs were found to be associated with two resistance-related modules. Among them, 463 DEGs related to anthracnose resistance were considered resistance-related candidate genes. Nineteen candidate genes were coexpressed with three resistance genes, *Phvul.001G243600*, *Phvul.001G243700* and *Phvul.001G243800*. To further identify resistance genes, 46 candidate genes were selected for experimental validation using salicylic acid (SA) and methyl jasmonate (MeJA). The results indicated that 38 candidate genes that responded to SA/MeJA treatment may be involved in anthracnose resistance in common bean.

**Conclusions:**

This study identified 38 resistance-related candidate genes involved in the early response of common bean, and 19 resistance-related candidate genes were coexpressed with anthracnose resistance genes. This study identified putative resistance genes for further resistance genetic investigation and provides an important reference for anthracnose resistance breeding in common bean.

**Supplementary Information:**

The online version contains supplementary material available at 10.1186/s12864-024-10497-7.

## Background

Common bean (*Phaseolus vulgaris* L.) is one of the most important legumes for direct human consumption, especially in many developing countries [1]. The annual planting area of common bean worldwide reaches 34.8 million hectares, with a total yield of 27.45 million tons. In China, the planting area is 743,703 hectares per year, with a total yield of 1.3 million tons. However, common bean production can be drastically impaired by many diseases [2]. Anthracnose is a serious threat to plant growth and development and occurs widely in common bean production areas [3].

Anthracnose, caused by the fungal pathogen *Colletotrichum lindemuthianum* (Sacc. and Magnus) Briosi and Cavara, is one of the most widespread and economically important pathogens of common bean [4]. Anthracnose can lead to devastating damage, such as severe yield, seed and pod quality losses. In extreme cases, yield losses due to anthracnose may reach 100% [5]. The spread of anthracnose can be reduced through agricultural control measures, such as seed treatment, rational crop rotation and improved field management. However, the identification of disease-resistant germplasms and genetic resources is the most economical and effective way to ensure yield in the production of common bean [6].

Many anthracnose-resistant germplasm resources, such as Hongyundou [5], Honghuayundou [7], AC [4], Paloma [8], AND 277 [9], B09197 [10] and PMB0225 [11], have been identified and evaluated in previous studies. These germplasms have been used for improved breeding and disease resistance research. To date, more than 40 resistance quantitative trait loci (QTLs) or genes for anthracnose have been identified on nine chromosomes. The resistance locus *Co-1* is located at the end of Pv01 with a distal physical position of 50.0-50.5 Mb. Subsequent research revealed that numerous anthracnose resistance genes or allelic genes were present in the resistance locus *Co-1*, such as *Co-x*, *Co-1*^*HY*^, *Co-1*^*65−X*^, *Co-1*^*73−X*^, *Co-Pa*, *Co-1*^*4*^ and *Co-AC* [4, 5, 8, 9, 12–15]. The locus *Co-x* was finely mapped to a 58-kb region flanked by the markers P05 and K06 by using a recombinant inbred line (RIL) population crossed between Jaloee P558 and BAT93, and eight candidate genes were identified in this region [13]. The locus *Co-Pa* is located in a 390-kb region flanked by the SNP markers SS82 and SS83, and nine candidate genes related to resistance were found [8]. The locus *Co-1*^*HY*^ was mapped to a 46 kb region on chromosome Pv01 via QTL mapping based on an RIL population cross between the anthracnose-resistant genotype Hongyundou and the susceptible genotype Jingdou, and four candidate resistance genes were identified [5]. These three loci, *Co-x*, *Co-Pa* and *Co-1*^*HY*^, have overlapping regions that include four candidate resistance genes: *Phvul.001G243500*, *Phvul.001G243600*, *Phvul.001G243700* and *Phvul.001G243800*. These four genes may have functions related to anthracnose resistance in common bean [13].

Transcriptome analysis is a common method for studying pathogen resistance-related genes in plants [16]. In maize, transcriptome analysis was performed using the plant infected by a low-virulence strain and a high-virulence strain of *Rhizoctonia solani*, and 388 potential key genes involved in the response to *R. solani* were identified, providing resistance genes for defense against banded leaf and sheath blight (BLSB) [17]. In cowpea, 103 candidate genes related to for root-knot nematode-mediated resistance were identified via differentially expressed gene (DEG) analysis, identifying novel potential resistance-related genes for further investigation in cowpea breeding [18]. In mungbean, 27 candidate genes involved in Fusarium wilt resistance were identified using Zheng8-4 and Zheng8-20 [19]. By combining DEG analysis and weighted gene coexpression network analysis (WGCNA) in common bean, a total of 139 DEGs were identified to be involved in common bean blight (CBB) resistance [20]. Comparative transcriptomic analyses were also carried out for anthracnose resistance in common bean. Padder et al. identified 3,250 DEGs in response to anthracnose race 73, and three DEGs were identified in the anthracnose-resistant locus *Co-1* [21]. Jurado et al. identified 23 DEGs in response to race 38 of *C. lindemuthianum* in the genotype carrying the *Co-2* gene [22]. These studies provided an understanding of anthracnose resistance at the transcriptomic level, but the available data on the resistance mechanism of common bean are limited.

Salicylic acid (SA), a phytohormone, regulates plant physiological and metabolic processes and induces plant resistance through various pathways [23]. Studies have shown that treatment with exogenous SA significantly increases the expression of relevant disease resistance genes in plants [24]. In banana, exogenous SA significantly increased the expression of key genes, including *PAL* and *SK*, involved in the SA synthesis pathway and the signaling pathway transcription factors *NPR1*, *TGA* and *PR1* [25]. Jasmonic acid (JA), a damage-related phytohormone and signaling molecule, is widely present in plants. Exogenous application of JA/MeJA induces the expression of resistance-related genes, leading to the production of resistance proteins and secondary substances and the formation of defense structures [26]. The application of MeJA to two JA-deficient mutants improved rice blast fungus resistance in rice, and the expression of JA-regulated resistance-related genes, such as *OsBBTI2* and *OsPR1a*, increased after fungal infection [27]. Therefore, the application of SA or JA/MeJA might induce plant resistance by influencing the expression of resistance genes.

In the present study, we selected two resistant genotypes, Hongyundou and Honghuayundou, and one susceptible genotype, Jingdou, for RNA sequencing. These genotypes were used in our previous studies for QTL mapping of anthracnose resistance loci [5, 28]. Differential expression analysis and WGCNA were conducted to identify anthracnose resistance-related genes involved in the early response of common bean. This study provides an important reference for the genetic research of anthracnose resistance in common bean.

## Methods

### Plant material and in planta inoculations

The two resistant genotypes Hongyundou and Honghuayundou and one susceptible genotype, Jingdou, were obtained from the National Crop Genebank of China (Beijing, China). For each genotype, five seeds were planted in paper cups and maintained at 20–22 °C, 95–100% humidity and a 12-h photoperiod in a climate-controlled chamber for 7–10 days. *C. lindemuthianum* race 81 was isolated from a naturally infected common bean cultivated in China [7]. It was routinely cultivated in Petri dishes containing potato dextrose agar (PDA) at 20 °C. Healthy plants were selected, and primary leaves were inoculated by spraying with an aqueous conidial suspension at a final concentration of 2.0 × 10^6^ spores/ml [5]. Leaf tissues of the three genotypes were collected before and after anthracnose pathogen inoculation at 0 and 6 hours post inoculation (hpi), with three sample replicates, for RNA-seq. All tests were repeated three times.

### RNA sequencing

A total of 18 leaf tissue samples from three genotypes collected before and after anthracnose pathogen inoculation were used for transcriptome analysis. The samples before pathogen inoculation were named R1 for Hongyundou, R2 for Honghuayundou, and S for Jingdou. After pathogen inoculation, the samples were named RP1 for Hongyundou, RP2 for Honghuayundou, and SP for Jingdou. Total RNA was extracted from the samples with TRIzol reagent (Invitrogen, CA, United States). Illumina sequencing was performed at Novogene, China. All samples were sequenced using the Illumina NovaSeq 6000 platform, and 150-bp paired-end reads were generated. Clean reads were obtained by removing reads containing adapters, reads containing poly-N and low-quality reads from the raw data. The Q20, Q30, and GC contents of the clean data were calculated. The reference genome model annotation files were downloaded from http://plants.ensembl.org/Phaseolus_vulgaris/Info/Index [29]. All genes were annotated by alignment to seven public databases, including the Nr, Nt, Pfam, Swiss-Prot protein, Clusters of Orthologous Groups for Eukaryotic Complete Genomes/Clusters of Orthologous Groups (KOG/COG), Gene Ontology (GO), and Kyoto Encyclopedia of Genes and Genomes (KEGG) databases. The transcript level of each gene was measured as fragments per kilobase of transcript sequence per millions of base pairs sequenced (FPKM).

### Differential expression analysis

Differential expression analysis was performed using the DESeq2 R package 1.20.0. The resulting *p* values were adjusted using the Benjamini and Hochberg method. Genes with an adjusted *p* value < 0.05 and a fold change > 2 were considered differentially expressed genes/transcripts. Bar and Venn diagrams were created with the R package. GO enrichment analysis of DEGs was performed with the clusterProfiler R package. GO terms with corrected *p* < 0.05 were considered significantly enriched. KEGG pathway enrichment analysis of the DEGs was implemented in KOBAS-i [30].

### Gene coexpression network analysis

Gene coexpression networks were constructed using the WGCNA package in R software (version 3.6.1). Unigenes with very low expression (FPKM < 1) in all samples were removed from this analysis to avoid the inclusion of spurious edges in the networks. Gene modules were identified using the parameters β value 20, minimum cluster size 30, and a merging threshold function of 0.25 (Fig. S1). Gene significance (GS) was used to correlate physiological data with gene expression data. The genes most significantly correlated with a WGCNA edge weight > 0.1 were visualized using Cytoscape 3.7.2 software.

### Treatments with SA and MeJA and qRT‒PCR analysis

Hongyundou and Jingdou were 7–10 days old, and primary leaves were treated with SA and MeJA (Sigma, Saint Louis, USA) by the spray method, with 0.02% Tween 80 used as the solvent. SA and MeJA were applied at a concentration of 2 mM/L [31]. Leaves were collected after 0 and 6 h and used for qRT‒PCR analysis, with three biological replicates. Total RNA was extracted from the samples using a Total RNA Isolation Kit (Nanjing Vazyme Biotech Co., China) according to the manufacturer’s instructions. cDNA was synthesized from each sample using *EasyScript®* First-Strand cDNA Synthesis SuperMix (TransGen Biotech, China). qRT‒PCR analysis of target genes in each resistant and susceptible time-series sample group was performed with an Applied Biosystems 7500 Real Time PCR system (Applied Biosystems, United States) using *TransStart®* Top Green qPCR SuperMix (TransGen Biotech, China) under the following conditions: initial denaturation at 94 °C for 30 s, 40 cycles of 5 s at 94 °C, 15 s at 58 °C, and 34 s at 72 °C. The specificity of the amplicons was validated by the final melting curve stage from 65 °C to 95 °C. Fluorescence intensity data were collected at the end of each cycle using the instrument’s software. The qRT‒PCR experiment was repeated with three biological replicates for each sample. Relative expression levels were calculated using the 2^−∆∆Ct^ method [19].

## Results

### Transcriptome sequencing and assembly

To investigate resistance-related genes involved in the early response to anthracnose, transcriptome sequencing was carried out using two resistant genotypes, Hongyundou and Honghuayundou, and one susceptible genotype, Jingdou, before and after inoculation (Fig. S2). All treatments were analyzed in three independent biological replicates. A total of 18 samples were sequenced, 0.81 billion clean data points were generated, and the mean length of each read was 150 bp (Table S1). The Q30% was greater than 95.74%, and the average GC content was approximately 43.98% (Table S1), indicating that the data were of high quality for further analysis. Based on the reference genome (Schmutz et al. 2014), an average of 93.35% of the clean reads were mapped in total, and 90.33% were mapped uniquely (Table S1).

A total of 27,197 genes were defined as known genes, and 1,637 transcripts were predicted as novel genes after filtration. The expression values of all genes were calculated from the FPKM values. Pearson’s correlation coefficients between the biological replicates of different samples revealed that the replicates were of high quality (Fig. S3). Principal component analysis (PCA) also indicated that the samples of the same treatment clustered together (Fig. S3), which confirmed the reliability and rationality of the experiment. A total of 20,509 genes with an average FPKM > 1 in at least one treatment were considered expressed genes and further analyzed.

### Identification of differentially expressed genes

**Fig. 1:**
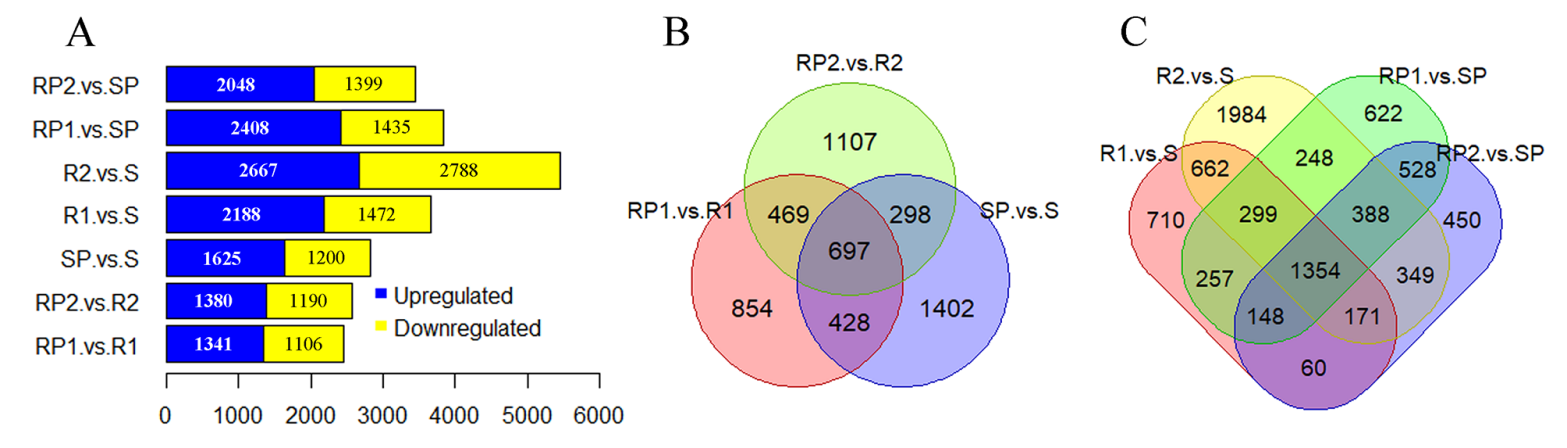
Overview of differentially expressed genes (DEGs). **A** Number of DEGs in each pair. Blue, upregulated DEGs; yellow, downregulated DEGs. **B** Venn diagram of the DEGs before and after inoculation. **C** Venn diagram of the DEGs between the resistant and susceptible lines

To identify DEGs responding to anthracnose, transcriptome comparisons were conducted before and after inoculation. By using a significance level of fold change > 2 and *p* value < 0.05, 2447, 2570 and 2,825 DEGs were found in RP1 vs. R1, RP2 vs. R2 and SP vs. S, respectively (Fig. 1A, B and Table S2). A total of 5254 DEGs were identified in response to pathogen inoculation (Table S2). To identify DEGs putatively involved in the resistance response, transcriptome comparisons were performed between the resistant and susceptible genotypes. There were 3660, 5455, 3843 and 3447 DEGs found in R1 vs. S, R2 vs. S, RP1 vs. SP and RP2 vs. SP, respectively (Fig. 1A and C and Table S2). A total of 8,229 DEGs were identified between the resistant and susceptible genotypes (Table S2). By combining the two DEG analyses above, 9,825 DEGs were selected for further investigation; these DEGs responded to pathogen infection and were differentially expressed between the resistant and susceptible genotypes.

### Coexpression network analysis of resistance-related DEGs

**Fig. 2:**
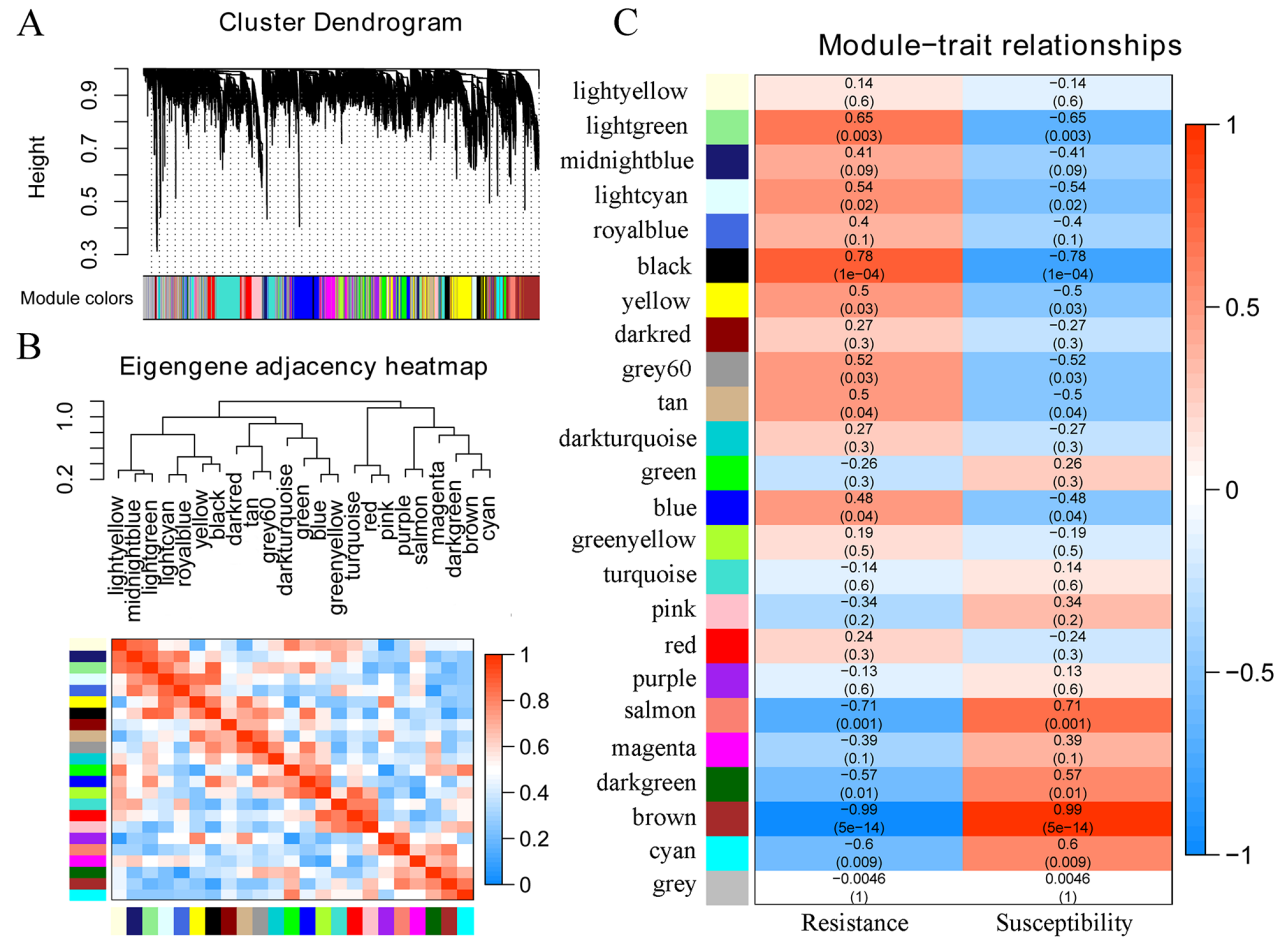
Weighted gene coexpression network analysis (WGCNA) of anthracnose resistance in common bean. **A** Hierarchical cluster diagram of coexpression modules according to WGCNA. **B** Gene coexpression modules showing the cluster dendrogram (above) and the heatmap for the correlation coefficient between the modules (below). **C** Module-trait relationships. Each cell contains the corresponding correlation and *p* value

**Fig. 3:**
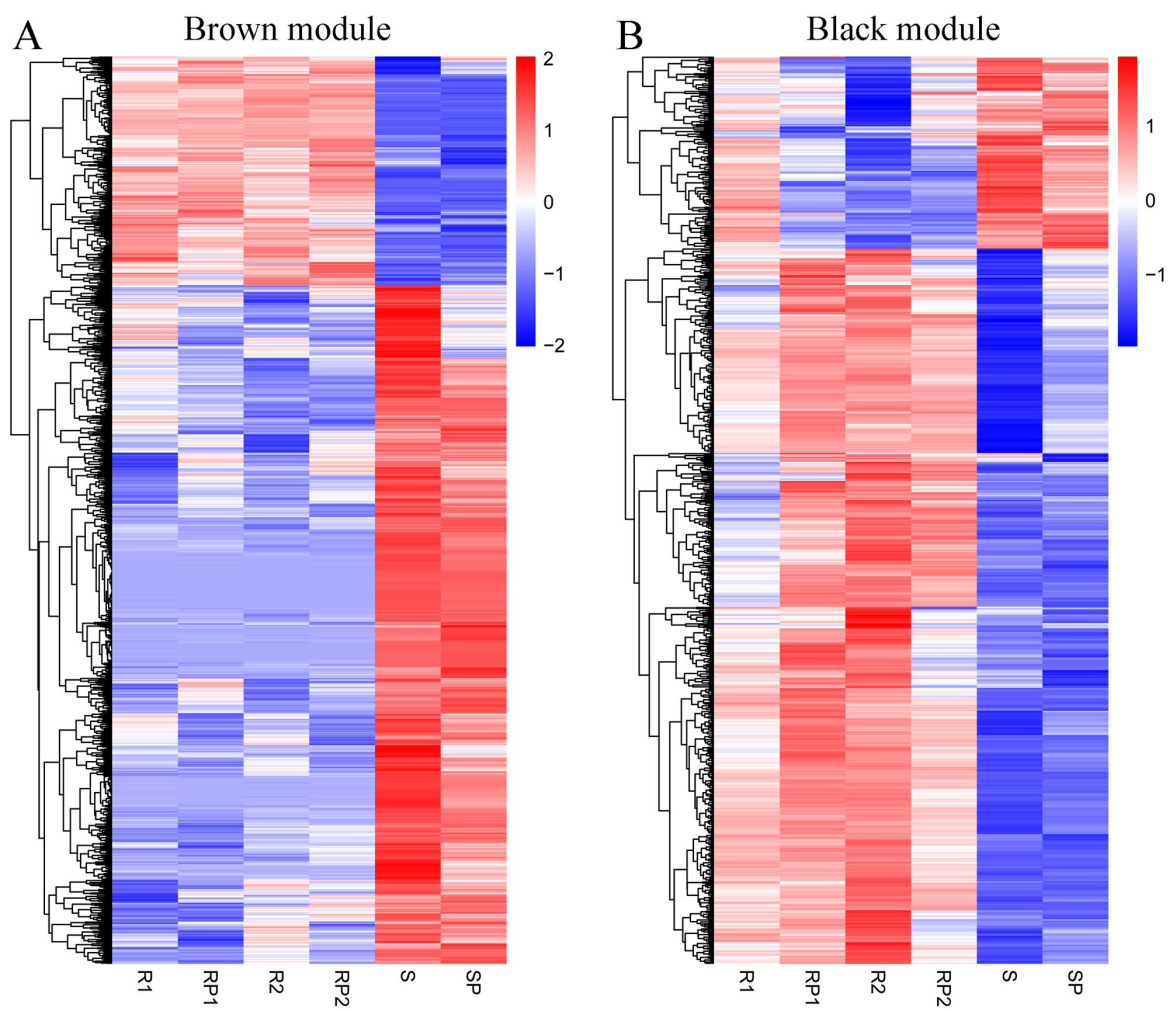
Expression pattern analyses of DEGs in the brown (**A**) and black (**B**) modules related to anthracnose resistance

To identify genes related to anthracnose resistance, we performed WGCNA of 20,509 genes. Several genes with similar expression profiles that belong to the same networks were clustered into the same WGCNA modules (Fig. 2A). When the power value (*β* value) was 20, a total of 24 WGCNA modules with gene numbers ranging from 85 to 2,465 were identified (Fig. 2B, S2 and Table S3). Correlations between modules and anthracnose resistance were then investigated. The brown (*R* = -0.99, *p* = 5e^− 14^) module and black (*R* = 0.78, *p* = 1e^− 4^) module correlated strongly with anthracnose resistance (Fig. 2C). A total of 1,953 genes clustered into the brown module, and 1,017 genes clustered into the black module (Table S3). The expression patterns of the genes in these two modules differed significantly between the resistant and susceptible genotypes (Fig. 3).

By combining the results of differential expression analysis and the coexpression network, 9,825 DEGs were distributed into 24 modules, with module sizes ranging from 9 DEGs to 1,395 DEGs (Table S3). There were 1,337 and 714 DEGs clustered into the resistance-related brown and black modules, respectively (Table S3). Therefore, a total of 2,051 DEGs were associated with the resistance-related module, which may have a function in anthracnose resistance.

### Identification of resistance-related candidate genes

**Fig. 4:**
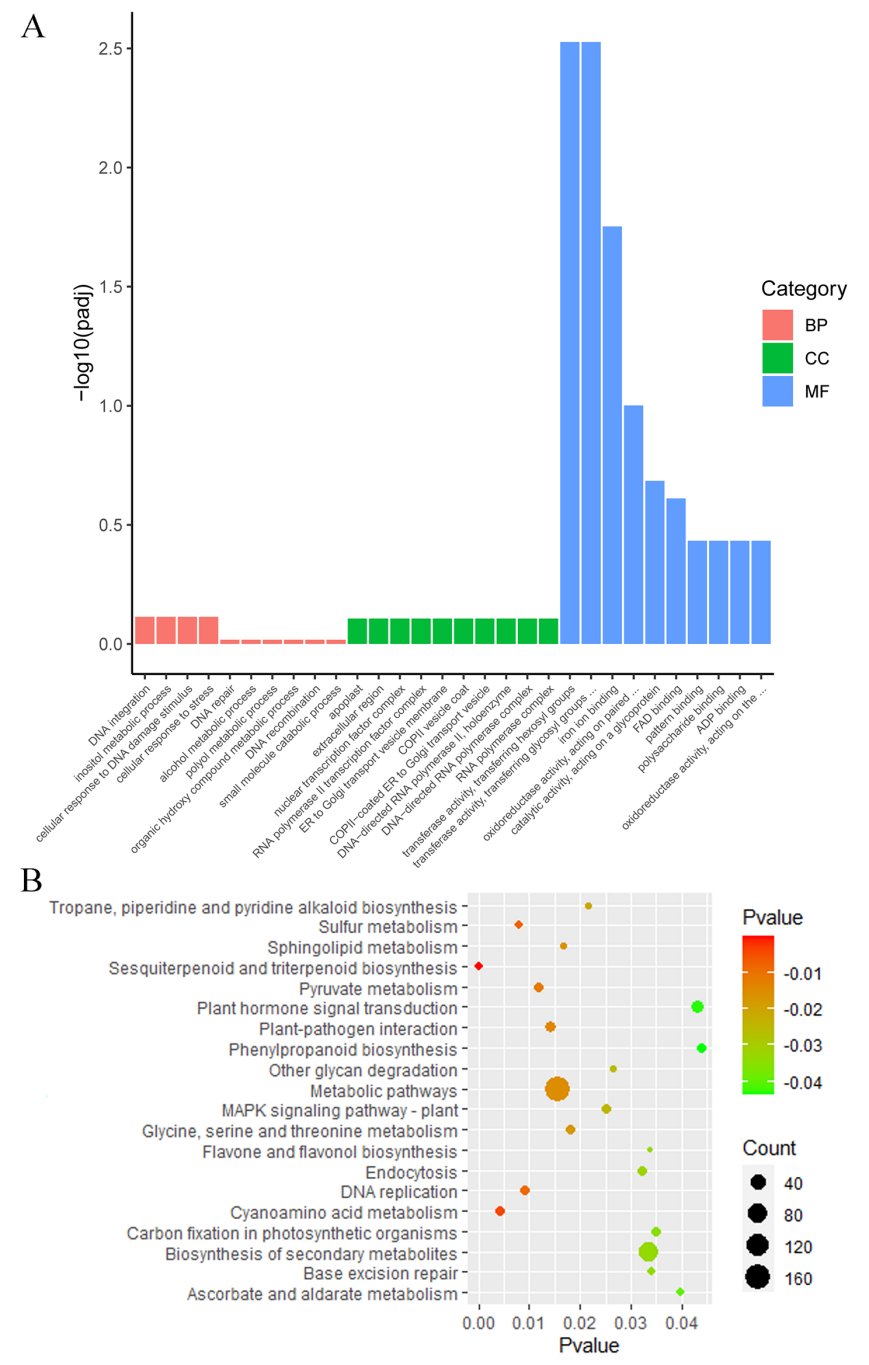
Functional analysis of 2051 resistance-related DEGs. **A** GO enrichment of DEGs. **B** KEGG enrichment of DEGs

Of the 2,051 DEGs, 96 genes were identified as resistance genes encoding disease resistance proteins. A total of 134 genes encoding protein kinases were obtained. Eighty-five genes were found to be transcription factor genes belonging to the WRKY, AP2, HLH, MYB, and bZIP gene families. (Table S4). To evaluate the possible functions of the 2,051 DEGs, GO enrichment was applied to identify functional categories. A total of 2,051 DEGs were differentially enriched in 776 terms (Fig. 4A and Table S5). Sixty-four of the DEGs were annotated with the terms “defense response” or “response to stress”, with 73 DEGs annotated as having oxidoreductase activity, indicating that these genes may respond to anthracnose resistance (Table S5). KEGG analyses were further conducted to investigate the functions of the 2,051 DEGs. These DEGs mapped to 114 pathways, and most DEGs were significantly enriched in “metabolic pathways” and “biosynthesis of secondary metabolites” (Fig. 4B and Table S6). We found that 56 DEGs clustered significantly into several pathogen response-related pathways, such as “plant‒pathogen interaction”, “MAPK signaling pathway”, “plant hormone signal transduction”, “phenylpropanoid biosynthesis” and “endocytosis” (Table S6). A total of 463 DEGs were considered resistance-related candidate genes that may be involved in anthracnose resistance.

**Fig. 5:**
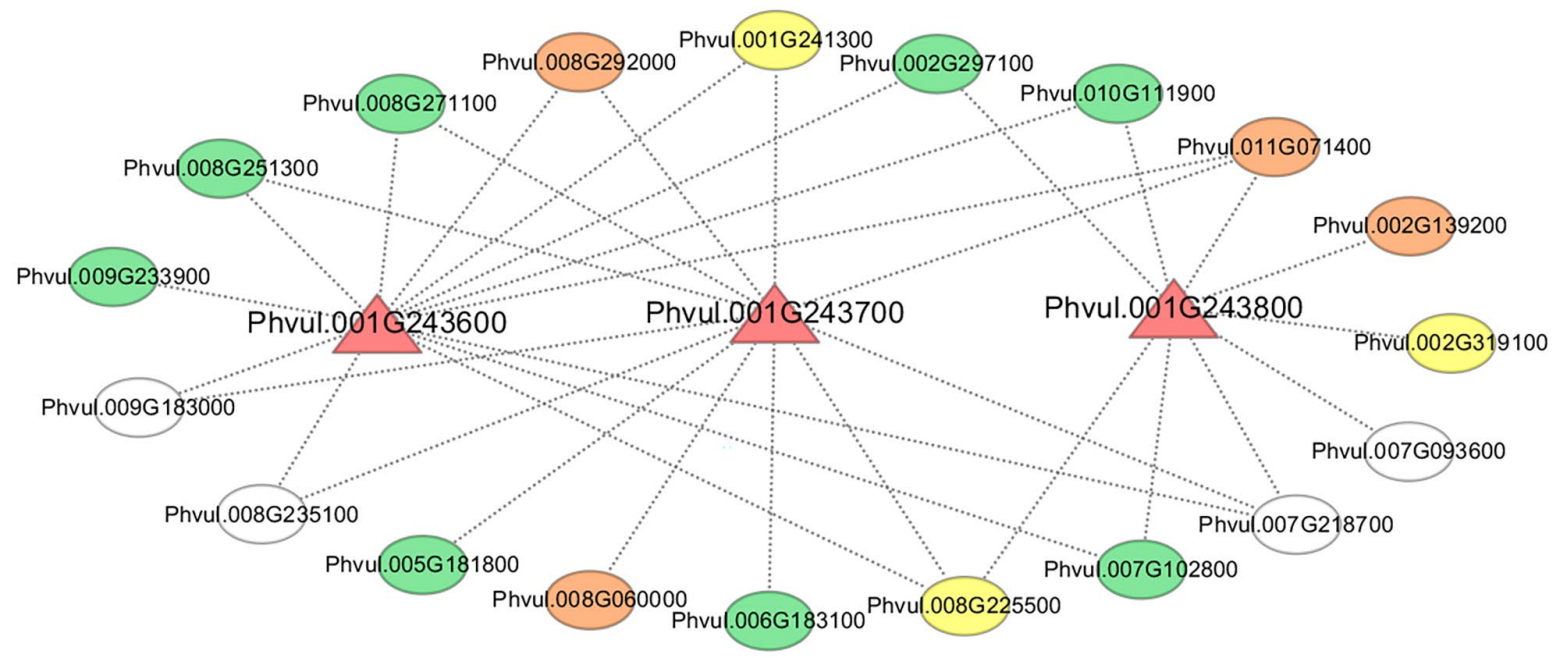
Cytoscape representation of genes coexpressed with *Phvul.001G243600*, *Phvul.001G243700* and *Phvul.001G243800*. The triangle nodes represent the target genes, and the ellipse nodes represent the coexpressed genes. The yellow, orange and green nodes represent resistance genes, protein kinase genes, and transcription factor family genes, respectively

Among the 463 DEGs, *Phvul.001G243600*, *Phvul.001G243700* and *Phvul.001G243800* were previously reported to be anthracnose resistance genes encoding serine/threonine protein kinases (Chen et al. 2017). We visualized the coexpression networks with *Phvul.001G243600*, *Phvul.001G243700* and *Phvul.001G243800*. Nineteen resistance-related candidate genes were coexpressed with the three genes, which may have similar functions (Fig. 5). These candidate genes included three R genes, four protein kinase genes and eight transcription factor family genes. *Phvul.001G241300*, *Phvul.008G225500*, *Phvul.007G093600* and *Phvul.007G218700* are related to the defense response; *Phvul.009G183000* and *Phvul.008G235100* are associated with “plant‒pathogen interaction”; and Phvul.008G251300 and Phvul.011G071400 are involved in the “MAPK signaling pathway” (Table S7). These candidate genes may contribute to studies of the resistance mechanism of anthracnose in common bean.

### Verification of resistance-related genes by SA and JA treatments

To further refine resistance-related genes, we screened 46 candidate genes from 463 DEGs and conducted experimental validation using qRT-PCR. The 46 candidate genes met at least two conditions: (1) R genes, protein kinase genes or transcription factor family genes; (2) genes associated with resistance-related functions and pathways; and (3) expression patterns that were significantly different between the resistant and susceptible genotypes. The expression trend of all candidate genes in qRT-PCR analysis was consistent with the RNA-seq data (Fig. S4), which supported the results generated by RNA-seq. To further verify candidate genes involved in plant resistance, the expression patterns of 46 candidate genes before and after SA or MeJA treatment were examined by qRT‒PCR.

**Fig. 6:**
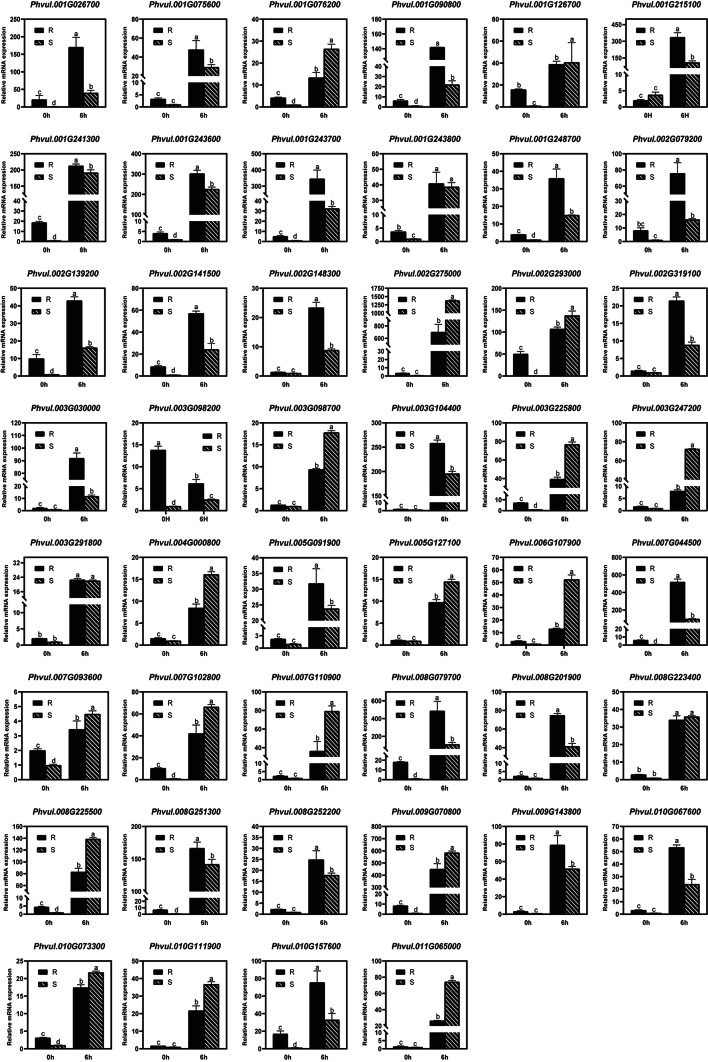
qRT‒PCR validation of resistance-related candidate genes after SA treatment in common bean. R, resistant genotype; S, susceptible genotype; h, hours after treatment

After SA treatment, the expression of 45 candidate genes was upregulated in both the resistant and susceptible genotypes, whereas that of one candidate gene was downregulated (Fig. 6 and Table S8). The expression pattern of 38 genes responding to SA was consistent with the pattern of genes responding to pathogen infection based on RNA-Seq (Table S8). Among these 38 genes, the expression of 19 candidates increased in the resistant genotype compared with the susceptible genotype after SA treatment, indicating that these genes may play positive roles in anthracnose resistance (Fig. 6). Furthermore, the expression of 17 candidate genes decreased more in the resistant genotype than in the susceptible genotype, indicating that these genes may have a negative regulatory effect on anthracnose resistance (Fig. 6). Additionally, the expression of two genes responding to SA did not differ significantly between the resistant and susceptible genotypes, suggesting that these genes may not be involved in anthracnose resistance in common bean (Fig. 6 and Table S8). Therefore, a total of 36 candidate genes that respond to SA treatment may be involved in anthracnose resistance in common bean.

**Fig. 7:**
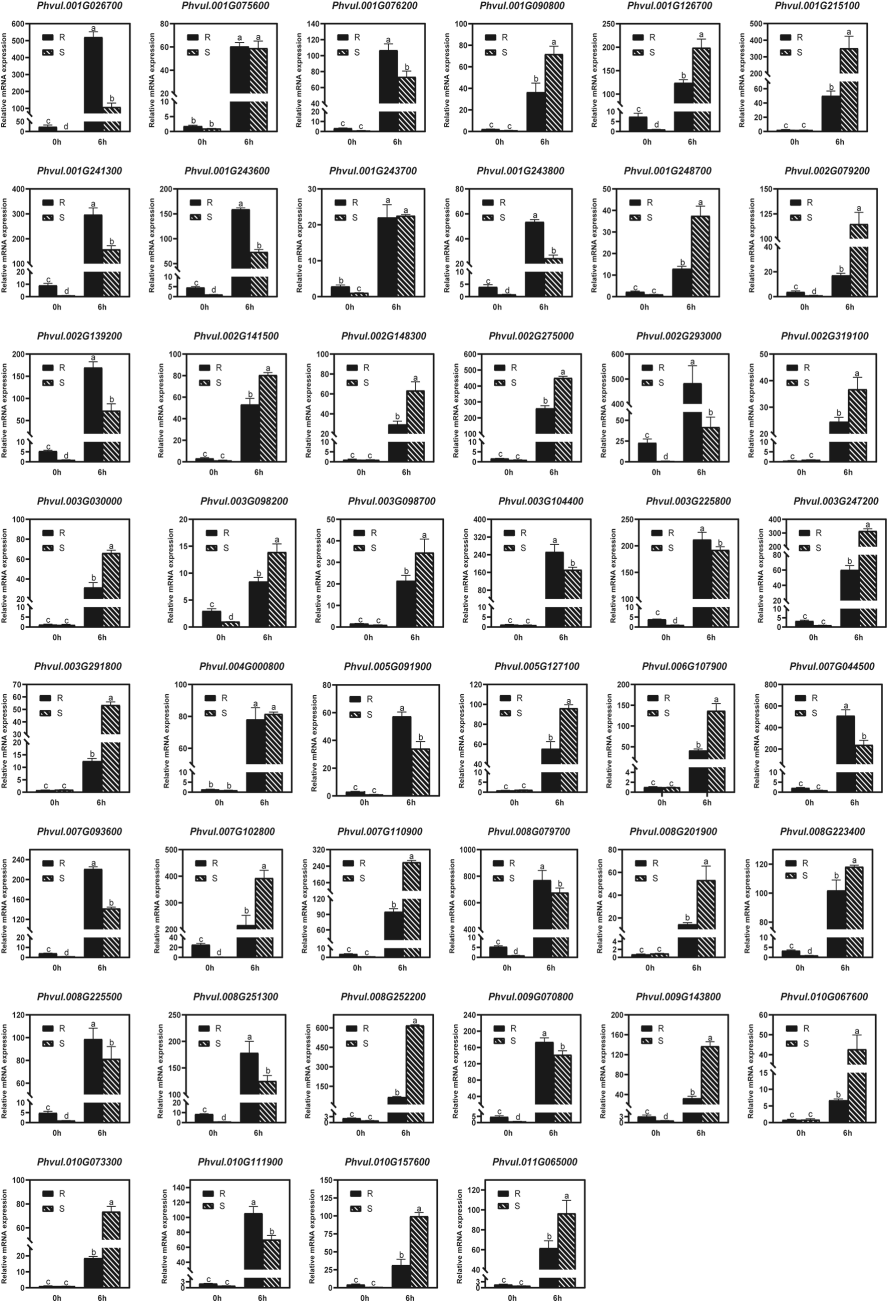
qRT‒PCR validation of resistance-related candidate genes after MeJA treatment in common bean. R, resistant genotype; S, susceptible genotype; h, hours after treatment

After MeJA treatment, the expression of 46 candidate genes significantly increased in both the resistant and susceptible genotypes; hence, these genes appear to respond to MeJA treatment (Fig. 7 and Table S8). The expression patterns of 37 genes responding to MeJA were consistent with the pattern of genes responding to pathogen infection according to the RNA-Seq data (Table S8). Among these 37 genes, the expression of 17 candidates increased in the resistant genotype compared with the susceptible genotype after MeJA treatment, suggesting their positive roles in anthracnose resistance (Fig. 7). The expression of 17 candidate genes decreased more in the resistant genotype than in the susceptible genotype, suggesting that these genes play negative roles in anthracnose resistance (Fig. 7). The expression of 3 genes responding to MeJA did not significantly differ between the resistant and susceptible genotypes, which indicates that these genes may not be related to anthracnose resistance in common bean (Fig. 7 and Table S8). Therefore, a total of 34 candidate genes that responded to MeJA treatment may be involved in anthracnose resistance in common bean.

In conclusion, a total of 38 resistance-related candidate genes responded to SA or MeJA, including ten genes encoding resistance (R) proteins, eleven genes encoding protein kinases, and ten genes belonging to transcription factor families (Table S9).

## Discussion

Anthracnose is one of the most important factors affecting common bean production worldwide. The resistant genotypes, Hongyundou and Honghuayundou, have been demonstrated to possess strong resistance to anthracnose race 81, and they were used for QTL mapping of anthracnose resistance loci *Co-1*^*HY*^ and *Co-F2533*, respectively [5, 28]. To further identify resistance-related genes involved in the same common bean-*C. lindemuthianum* interaction, these genotypes were selected for transcriptome analysis. Previous common bean - *C. lindemuthianum* interaction studies focused on gene expression beyond 24 hpi [21, 22, 32]. However, the early response of common bean to *C. lindemuthianum* has not been fully characterized. Therefore, we focused on the response of DEGs to *C. lindemuthianum* at 6 hpi. We hope these results will provide a reference for future studies on resistance mechanisms and anthracnose resistance breeding in common beans.

The SA and JA pathways are the main phytohormone signaling pathways related to plant resistance. Exogenous application of SA and JA can induce plant resistance by influencing the expression of resistance genes [24–27]. Therefore, DEGs were further verified using exogenous SA and JA in this study. We found that most candidate genes whose expression was upregulated in response to SA and JA (Figs. 6 and 7). After SA or JA treatment, the changes in gene expression differed between the resistant and susceptible genotypes (Figs. 6 and 7). Except for genes responding to SA or JA did not significantly differ between the resistant and susceptible genotypes, a total of 38 resistance-related genes were identified as key genes. The selected DEGs that responded to SA and JA may be involved in plant disease resistance.

The 38 resistance-related candidate genes included ten R genes, eleven protein kinase genes, and ten transcription factor family genes (Table S9). R genes play a crucial role in the regulatory process of plant disease resistance [33]. *Phvul.003G247200* encodes a coiled coil domain at the N-terminus (CC-NBS-LRR), and comparative analysis revealed that this gene is homologous to the *At5g66900* gene in *Arabidopsis thaliana*. *Phvul.001G075600*, *Phvul.001G076200*, *Phvul.003G030000* and *Phvul.010G073300* encode R proteins with protein kinase domains, suggesting that they may be involved in signal transduction during the resistance response to pathogens. *Phvul.001G241300* and *Phvul.008G225500* encoding nematode resistance HSPRO2-like proteins are involved in the defense response (Table S5). Transcription factors play important regulatory roles in plants in response to pathogen infection [34–38]. The WRKY transcription factor gene *Phvul.008G251300* was found to participate in the “MAPK signaling pathway”, while the bZIP transcription factor gene *Phvul.003G291800* was involved in “plant hormone signal transduction” (Table S9). These genes may be involved in regulating anthracnose resistance in common bean and need to be further explored.

In a previous study, four genes were mapped to the anthracnose resistance locus *Co-1*^*HY*^ [5]. Three of them, *Phvul.001G243600*, *Phvul.001G243700* and *Phvul.001G243800*, were also identified in this study. The three genes encoded serine/threonine-protein kinases and were expressed at significantly greater levels in the resistant genotype than in the susceptible genotype. In particular, *Phvul.001G243600* and *Phvul.001G243700* are more than 15-fold and 90-fold more highly expressed, respectively, in the resistant genotype than in the susceptible genotype [5]. We found that 19 resistance-related candidate genes were coexpressed with three genes, including three R genes, four protein kinase genes and eight transcription factor family genes. Among them, the R genes *Phvul.008G225500* and *Phvul.001G241300* were related to the defense response, and the protein kinase gene *Phvul.011G071400* and WRKY transcription factor gene *Phvul.008G251300* were involved in the “MAPK signaling pathway” (Fig. 5 and Table S7). These coexpressed candidate genes are helpful for further research on the resistance mechanism of anthracnose in common bean. The *Co-1*^*HY*^ locus was colocalizes with the *Co-1* resistance locus [5]. Padder et al. conducted transcriptome analysis using resistant and susceptible NILs at 24, 72 and 96 hpi, and six DEGs were identified in the *Co-1* genomic region [21]. Among these six DEGs, *Phvul.001G243700* and *Phvul.001G241300* were identified as resistance-related candidate genes in our study. *Phvul.001G241300* encoded the nematode resistance protein HSPRO2. HSPRO2 of *Arabidopsis* responded to *Pseudomonas syringae* infection, indicating a role for HSPRO2 in *Arabidopsis* basal resistance against the bacterial pathogen [34]. We found that *Phvul.001G241300* was induced by SA and JA and was coexpressed with *Phvul.001G243600* and *Phvul.001G243700* (Figs. 5, 6 and 7). *Phvul.001G241300* possibly interacted with *Phvul.001G243600* and *Phvul.001G243700* and may be involved in anthracnose resistance in common bean. Moreover, the candidate genes *Phvul.002G293000* and *Phvul.003G247200* were also shown to respond to *C. lindemuthianum* in BAT93 [32], indicating their putative roles in anthracnose resistance. Additionally, we explored DEGs at other anthracnose resistance-related loci. However, we did not identify any DEGs at other loci, possibly because differences between our study and previous studies in terms of genotypes, *C. lindemuthianum* races and inoculation time. Different genotypes may have different anthracnose resistance mechanisms in common bean.

## Conclusions

In conclusion, a total of 38 resistance-related candidate genes were identified in the early response of common bean. Moreover, 19 resistance-related candidate genes were coexpressed with three anthracnose resistance genes. These results provide potential anthracnose resistance genes for further investigation and genetic resources for anthracnose resistance breeding in common bean.

### Electronic supplementary material

Below is the link to the electronic supplementary material.

Supplementary Material 1

Supplementary Material 2

## Data Availability

All transcriptome data were stored on the NCBI SRA platform with accession number PRJNA1113422.

## Abbreviations

DEGs: Differentially expressed genes
JA: Jasmonic acid
MeJA: Methyl jasmonate acid
QTL: Quantitative trait locus
RIL: Recombinant inbred line
R protein: Resistance protein
SA: Salicylic acid
WGCNA: Weighted gene coexpression network analysis
