# Supplementary material for: Global transcriptome analysis reveals resistance genes in the early response of common bean (Phaseolus vulgaris L.) to Colletotrichum lindemuthianum
Source: BMC Genomics. 2024 Jun 10;25:579. doi: 10.1186/s12864-024-10497-7 (PMC11165746; doi:10.1186/s12864-024-10497-7)
Supplement: Supplementary file 2 — Supplementary Material 2 [file 12864_2024_10497_MOESM2_ESM.docx]

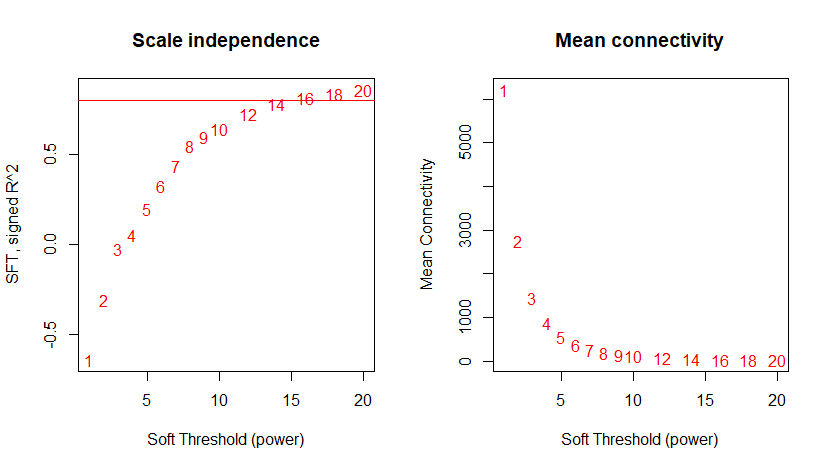


**Fig. S1** Analysis of network topology for various soft-thresholding powers. The left panel shows the scale-free fit index(y-axis) as a function of the soft-thresholding power(x-axis). The right panel shows mean connectivity (degree, y-axis) as a function of the soft-thresholding power(x-axis).


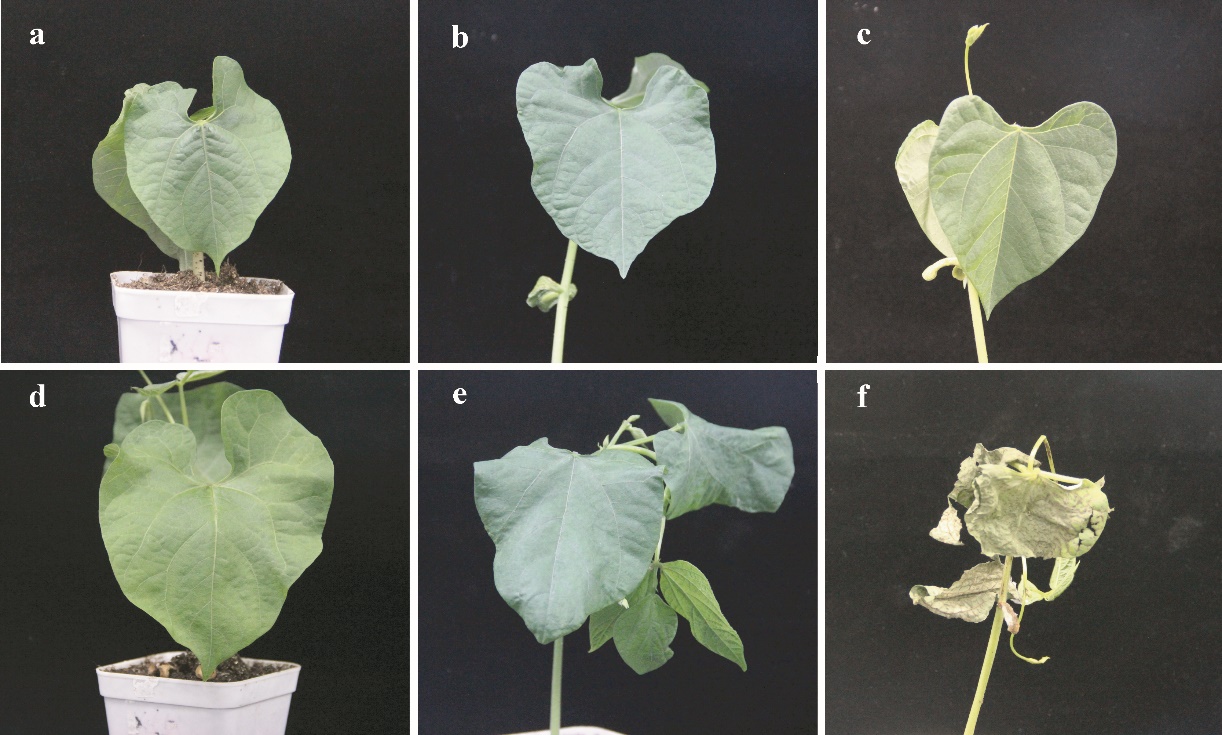


**Fig. S2** Symptoms before and after inoculation with *Colletotrichum lindemuthianum* in Hongyundou (a,d), Honghuayundou (b, e) and Jingdou (c, f). (a-c) Phenotypes before inoculation. (d-f) Phenotypes at 7 days after inoculation.


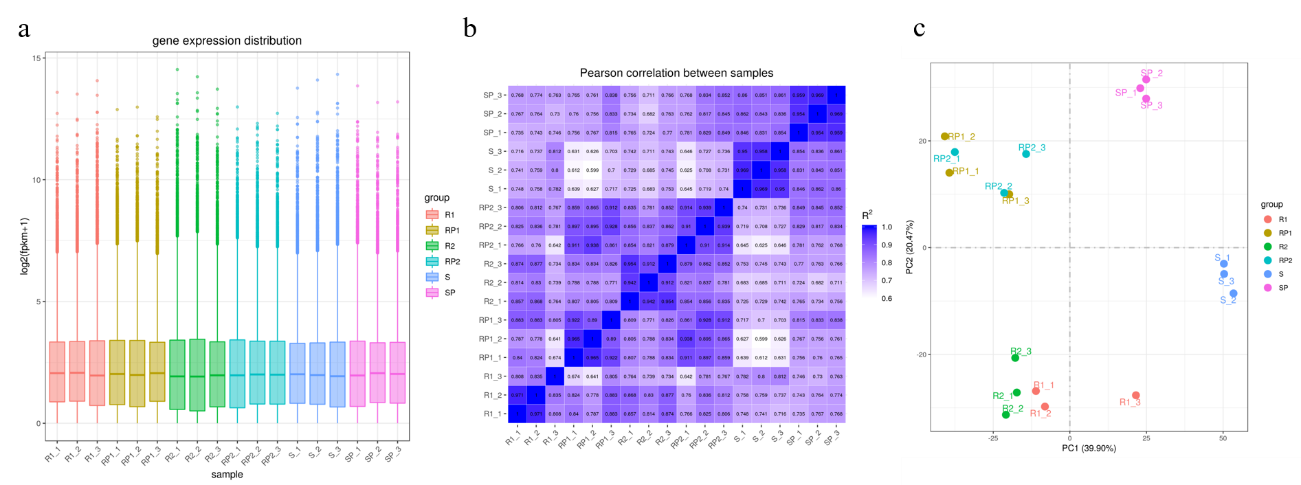


**Fig. S3** Sample gene expression distribution and correlation analysis. (a) Overall distribution of sample gene expression - box plot. (b) Sample pearson correlation analysis. (c) PCA principal component analysis.


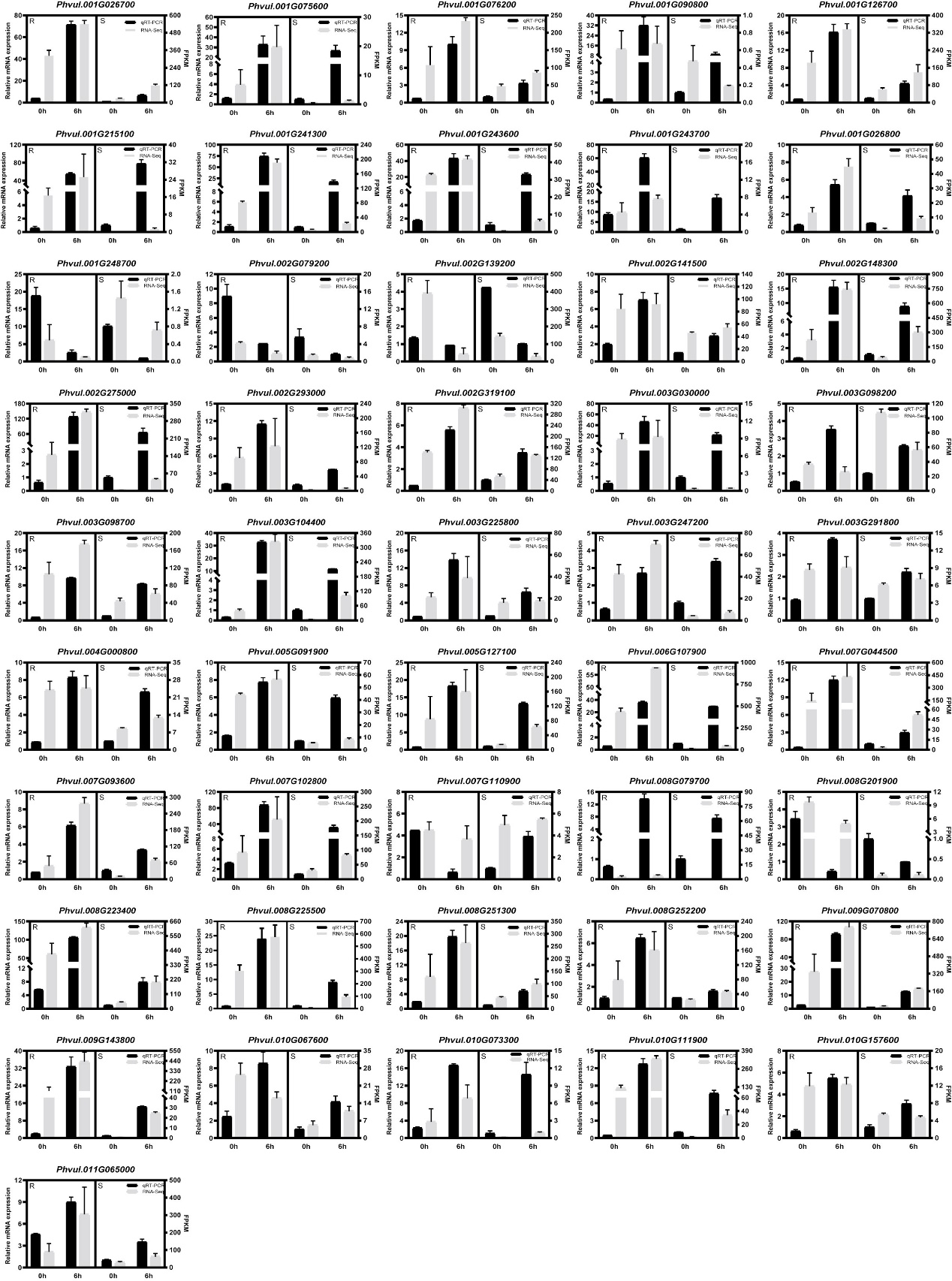


**Fig. S4** qRT-PCR validation of 46 candidate genes at 0 and 6 hours post inoculation. R, resistant genotype Hongyundou; S, susceptible genotype Jingdou.
